# Periocular Skin Warming Promotes Sleep Onset Through Heat Dissipation From Distal Skin in Patients With Insomnia Disorder

**DOI:** 10.3389/fpsyt.2022.844958

**Published:** 2022-05-04

**Authors:** Tomohisa Ichiba, Aoi Kawamura, Kentaro Nagao, Yuichi Kurumai, Akio Fujii, Atsushi Yoshimura, Takuya Yoshiike, Kenichi Kuriyama

**Affiliations:** ^1^Personal Health Care Laboratory, Kao Corporation, Tokyo, Japan; ^2^Department of Psychiatry, Shiga University of Medical Science, Otsu, Japan; ^3^Department of Sleep-Wake Disorders, National Center of Neurology and Psychiatry, National Institute of Mental Health, Kodaira, Japan

**Keywords:** insomnia disorder, heat dissipation, periocular skin warming, skin temperature, sleep onset, thermoregualtion

## Abstract

**Study Objectives:**

Periocular skin warming before bedtime has been demonstrated to improve subjective sleep initiation in healthy adults with sleep difficulties scored six or higher in the Pittsburgh Sleep Questionnaire Index. This study aimed to investigate the effects of periocular skin warming on sleep initiation and thermoregulation processes in patients with insomnia disorder.

**Methods:**

Participants included those with sleep difficulty (*n* = 22) and those with insomnia disorder (*n* = 16). Individuals from both groups were assessed at baseline (habitual sleep-wake schedule) and after two intervention conditions (use of a warming eye mask or a sham eye mask before habitual bedtime). The subjective and electroencephalographic sleep onset latency, along with proximal and distal skin temperature after periocular skin warming, were evaluated.

**Results:**

Periocular skin warming reduced objective sleep onset latency in independently of the group. Foot temperature and foot-proximal temperature gradient after getting into bed increased with periocular skin warming in independently of the group. However, the increase in hand temperature was observed only in the insomnia disorder group. Periocular skin warming also increased the normalized high frequency component of heart rate variability in independently of the group. The reduction of objective sleep onset latency was strongly associated with heat dissipation from the foot skin region.

**Conclusion:**

These results suggest that periocular skin warming promotes sleep initiation by enhancing heat dissipation from the distal skin regions in individuals with sleep difficulty and insomnia disorder. Periocular skin warming could thus be a novel non-pharmacological therapy for insomnia disorder.

## Introduction

Approximately 20% of adults experience difficulty sleeping (1, 2), and 10–15% of them meet the diagnostic criteria for insomnia disorder (1, 2). According to the Diagnostic and Statistical Manual of Mental Disorders (DSM-5), insomnia disorder is characterized by three types of sleep difficulty (difficulty initiating sleep, difficulty maintaining sleep, and early morning awakenings) along with consequent daytime dysfunctions such as fatigue or excessive daytime sleepiness. Difficulty initiating sleep is the most common sleep-related symptom (9.8%) when compared with the others (7.1%: difficulty maintaining sleep and 6.7%: early morning wakening), and these symptoms are often comorbid with each other in the Japanese population (3). Both pharmacotherapy and cognitive behavioral therapy for insomnia have been proven to be effective for treating difficulty initiating sleep (4), with the latter treatment being recommended as the first-line treatment worldwide (5, 6). However, while cognitive behavioral therapy for insomnia can improve subjective complaints of difficulty initiating sleep, it seems ineffective in improving objective sleep onset latency (SOL) (7, 8). This could be the case because cognitive behavioral therapy is primarily used to treat subjective symptoms. Thus, a novel non-pharmacological therapy that can improve both subjective complaints and objective SOL while maintaining safety and cost efficiency should be developed according to the physiological basis of insomnia.

Various body temperature manipulations, which can change thermoregulatory responses such as increasing distal heat dissipation, have been found to promote sleep onset (9–13). For instance, body heating *via* hot bath immersion 1–2 h before bedtime has been reported to reduce SOL (9–11). It has been speculated that the facilitative effects of whole-body warming on sleep onset might be achieved *via* acceleration of heat dissipation from the body after a temporary increment of core body temperature (CBT) (10, 12, 13). Although a rapid decrease in CBT seems to facilitate sleep onset, foot warming, which does not raise CBT, has also been demonstrated to reduce SOL (13). Furthermore, the distal-proximal skin temperature gradient (DPG) between the distal (hands and feet) and proximal (e.g., thighs, infraclavicular region, stomach) skin regions has been reported to be negatively correlated with SOL more strongly than CBT (14). Based on these findings, it can be hypothesized that distal skin warming is crucial for promoting sleep onset.

Recently, we developed a disposable heat and steam generating sheet (HSG-sheet) that can safely and easily manipulate periocular skin temperature (15–17) in the home environment. In a preliminary study, we demonstrated that periocular skin warming improves subjective sleep initiation in healthy individuals with mild difficulty falling asleep (15). In addition, periocular skin warming during the daytime in healthy men has been demonstrated to increase the distal skin (hands and foot) temperature and DPG, resulting in the promotion of sleep onset in a laboratory environment (16, 17). Considering that periocular skin warming has similar thermoregulatory effects on sleep as distal skin warming, periocular skin warming might be safely and easily employed at home as a treatment option for insomnia disorder. However, the effects of periocular skin warming on nocturnal sleep onset in patients with insomnia disorder remains unknown.

In the present study, we asked patients with insomnia disorder to wear an eye mask containing an HSG-sheet before their habitual bedtime in their home environment to determine the effect of periocular skin warming on sleep onset and thermoregulation processes. We monitored electroencephalography (EEG)-based SOL and proximal and distal skin temperature during nocturnal sleep.

## Materials and Methods

### Participants

Participants who have met at least one of the inclusion criteria, namely, (1) difficulties initiating sleep, (2) difficulties maintaining sleep, or (3) waking up earlier than desired within the last 6 months, and who were aged between 45 and 70 years were recruited through online advertisements from May 1 to November 21, 2018. The exclusion criteria were the following: (1) Presence of pre-existing psychiatric and sleep disorders other than insomnia disorder (18), and (2) insomnia possibly caused by medical conditions (i.e., cardiovascular disease, neuropsychiatric disorder, allergy disease, or renal urological diseases), drugs, or other substances.

**FIGURE 1:**
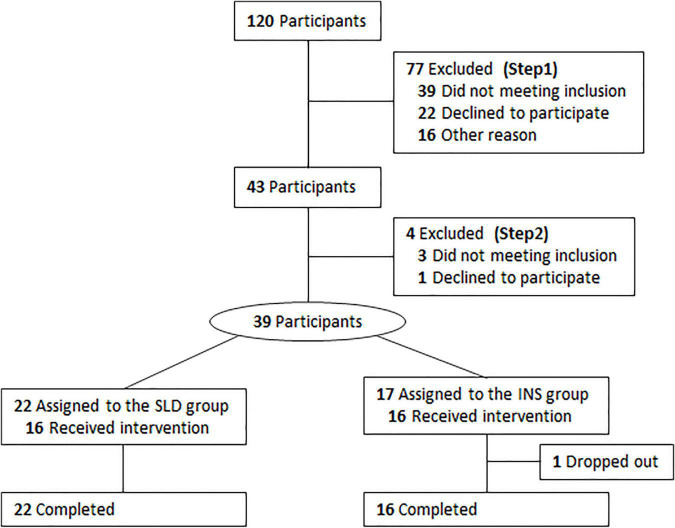
Flowchart diagram of the study protocol from entry to completion. SLD, sleep difficulty; INS, insomnia disorder.

Participants who responded to the advertisements underwent a two-step screening process, which consisted of (1) a telephonic screening and (2) a medical interview by well-trained psychiatrists to ensure that participants met the inclusion and exclusion criteria (Figure 1). A total of 120 participants were screened by telephone, and 77 participants were excluded (step 1). The remaining 43 participants completed the Japanese version of the Pittsburgh Sleep Quality Index (PSQI) (19), the Sheehan Disability Scale (SDISS) (20), the 9-item Patient Health Questionnaire (PHQ-9) (21, 22), and the State-Trait Anxiety Inventory (STAI) (23), after which they underwent step 2. After excluding 4 more participants who did not meet the appropriate criteria or who declined to participate, 39 participants were ultimately included. They completed a clinical interview conducted by well-trained psychiatrists according to the DSM-5 criteria for the diagnosis of insomnia disorder (18). Participants who met the criteria for insomnia disorder were assigned to the insomnia disorder (INS) group (*n* = 17). Participants who did not meet at least one of the following criteria were assigned to the sleep difficulty (SLD) group (*n* = 22): (1) Significant distress or interference with personal functioning in daily living caused by the sleep difficulty; (2) occurrence of sleep difficulty at least three times a week; (3) presence of sleep difficulty for at least 3 months. One participant in the INS group dropped out during the baseline session. Ultimately, 22 participants in the SLD group and 16 participants in the INS group completed the whole study protocol and were included in the final analysis.

All participants provided written informed consent after receiving a detailed explanation of the experiment. The study was conducted according to the tenets of the Declaration of Helsinki. This study was approved by the Ethics Committee of Shiga University of Medical Science.

### Experimental Protocol

**FIGURE 2:**
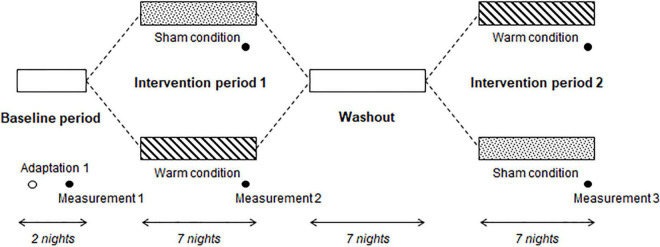
Experimental protocol. Participants maintained their habitual sleep-wake schedule during the Baseline period. During Intervention period 1, participants were allocated to the sham condition (wore a sham eye mask before their usual bedtime) or warm condition (wore a warming eye mask before their usual bedtime). After the Washout, participants wore the other eye mask (i.e., that they had not worn during Intervention period 1) during Intervention period 2. In the Baseline period, electroencephalography, skin temperature, and electrocardiography were measured for 2 consecutive nights. The same measurements were performed at the end of Intervention periods 1 and 2.

After participant screening, all experimental procedures were performed at the participants’ homes. First, participants were asked to maintain their habitual sleep–wake schedule for 2 days (baseline period), and subsequently underwent two intervention periods, separated by 1 week. One intervention period was set as the “warm condition” and the other was set as the “sham condition” (Figure 2). In the first intervention period, participants were instructed to wear a warming eye mask or a sham eye mask just before their habitual bedtime. The participants could not distinguish between a warming eye mask and a sham eye mask based on their appearance. In the second intervention period, participants were instructed to wear an eye mask (warming or sham) different from that employed during the first intervention period. Each intervention period lasted 7 consecutive nights. To measure sleep and physiological parameters, participants were instructed to use portable EEG and electrocardiography (ECG) devices and wireless skin temperature sensors at the final nights of the baseline and intervention periods. Throughout the entire experimental period, participants were asked to maintain the dose and type of their regular medication, and to refrain from consuming alcohol, smoking, and ingesting caffeinated beverages during the evening. They were also instructed to finish exercising and bathing/showering 1 h before their bedtime.

### Eye Masks

The warming eye mask with an HSG-sheet and the sham eye mask were manufactured with the same non-woven fabric sheets. A disposable eye mask with an HSG-sheet, which provided moist heat through the chemical reaction of iron, water, and oxygen, was confirmed to gradually increase periocular skin temperature to 38°C within 5 min and maintain it at 38–40°C for approximately 20 min (16). The sham eye mask, which had an inactive HSG-sheet, did not change the periocular skin temperature. Details of the changes in periocular skin temperature that occur while using both eye masks have been described elsewhere (16). The eye masks were custom-made by Kao Corporation (Tokyo, Japan) as a prototype device to be used in the present study.

### Measurements of Objective and Subjective Sleep Onset Latency

Objective SOL was measured in the participants’ home using a single-channel portable EEG device (SLEEP SCOPE; SleepWell Co., Osaka, Japan) at a sampling rate of 128 Hz (15, 24). Before bedtime, participants were instructed to place disposable Ag/AgCl surface electrodes at the median frontal region, referenced by a right or left mastoid electrode. The raw signals were later analyzed off-line. The EEG-based objective SOL was defined as the time interval between the time into bed and the onset of the first ten continuous 30-s epochs of sleep, as defined by previous criteria (15, 24). To measure subjective SOL, participants were instructed to fill a recording sheet in the mornings.

### Measurements of Skin Temperature

Skin temperature was continuously measured using wireless temperature sensors (iButton ^®^ DS1920, Maxim Integrated, San Jose, CA, United States) at a sampling rate of 1/60 Hz with a resolution of 0.0625°C, which allows reliable and valid measurements of human skin temperature (25, 26). Before bedtime, participants were instructed to fix the temperature sensors on the right side of the infraclavicular region, the foot (the middle of the dorsal side), and the hand (the middle of the dorsal side) using thin, air-permeable, adhesive surgical tape (Fixomull stretch, BSN Medical, Hamburg, Germany), as described in a previous study (27). All skin temperature data were measured from the time in bed and averaged for 5-min bins, except for excessively low temperatures. The right infraclavicular skin temperature was defined as the proximal skin temperature. The foot and hand skin temperatures were defined as the distal skin temperature. The DPG was calculated by subtracting the proximal skin temperature from the distal skin temperature in the foot or hand (14).

### Measurements of Heart Rate Variability

ECG was recorded using a Hexoskin smart shirt (Carre Technologies Inc., Montreal, Canada), which is a function shirt with built-in cardiac sensors in the thorax and abdomen that record at a sampling rate of 256 Hz. The heart rate variability (HRV) analyses were performed according to previous studies (16, 28). The R-R interval was detected from ECG recordings, and spectral analysis of the R-R interval for the 5-min period was performed using a Fast-Fourier Transform to obtain low-frequency (LF; 0.04–0.15 Hz) and high-frequency (HF; 0.15–0.40 Hz) powers. Furthermore, normalized HF [normHF = HF/(LF + HF)] were estimated. The normHF value was used as an index of cardiac parasympathetic activity. These analyses were performed using the MATLAB software package (Math Works Inc., Natick, MA, United States) and its signal processing toolbox.

### Sample Size

The sample size was calculated by using the G*Power version 3.1. We used an analysis of variance (ANOVA) as fixed effects with alpha level of 0.05 and power of 0.80. A sample size of 34 participants was planned. Finally, we used a total sample size of 40, taking into account possible dropouts and missing data.

### Statistical Analysis

All values are expressed as mean ± standard error of the mean (SEM). Comparisons of demographic and clinical variables between the SLD and INS groups were performed using a Student’s *t*-test or a chi-square test.

The data distribution and equality of variances of the objective and subjective SOL data were verified using the Shapiro-Wilk normality tests and Levene’s tests, respectively. Then, the objective and subjective SOL values were compared between the sham and warm conditions using two-way ANOVA with “group” and “condition” as fixed effects. The hand and foot skin temperature with respective DPGs over the initial 30 min (6 × 5-min bins) after the time into bed were analyzed using a mixed-model three-way ANOVA with “group” and “condition” as fixed effects. The R-R interval, HF, and normHF for the initial 5 min after the time into bed were also analyzed using a two-way ANOVA with “group” and “condition” effects. *Post hoc* comparisons were performed using Bonferroni’s correction test with an adjusted *p*-value. Furthermore, backward stepwise multiple regression analysis was conducted to identify the contribution of thermoregulatory variables and HRV to objective or subjective SOLs. The level of significance was set at 5% (two-sided test). All statistical analyses were performed using IBM SPSS Statistics 25 (IBM, Chicago, IL, United States).

## Results

### Demographic and Clinical Characteristics of the Sleep Difficulty and Insomnia Disorder Groups

The demographic and clinical characteristics of the two groups are shown in Table 1. There was no significant difference between the two groups in age, sex, body mass index, or current use of drugs that could affect sleep (included diazepam and brotizolam) or autonomic nervous function (included amlodipine besilate, telmisartan, and atenolol). Consistent with the group characteristics, the PSQI, PHQ-9, and SDISS scores were significantly higher in the INS group than in the SLD group. There was no significant difference between the two groups in the STAI (*p* = 0.086). At baseline, there was no significant difference in the EEG-based objective and subjective SOL between the SLD and INS groups.

**TABLE 1:** The participant’s demographic and clinical characteristics.

Characteristic	SLD (*n* = 22)	INS (*n* = 16)	*p*-value
Age (years)	56.1 (1.5)	55.9 (1.9)	0.913
Sex; male/female (%)	36.4/63.6	50.0/50.0	0.401
Body mass index (kg/m^2^)	22.2 (0.8)	21.7 (1.1)	0.706
**Current drug use**			
Hypnotic (%)	0	6.7	0.235
Sympathomimetics (%)	10.0	14.3	0.735
PSQI	8.4 (0.4)	10.3 (0.6)	**0.004**
PHQ-9	3.7 (0.5)	5.6 (0.6)	**0.028**
SDISS	1.0 (0.4)	4.3 (1.2)	**0.007**
STAI trait anxiety	38.5 (2.1)	44.3 (2.4)	0.086
Objective SOL (min)	34.4 (10.8)	24.7 (4.6)	0.462
Subjective SOL (min)	28.7 (5.2)	45.7 (11.4)	0.061

*All values are expressed as the mean (SEM). Objective and subjective SOLs were measured at baseline.*

*SLD, sleep difficulties; INS, insomnia disorder; PSQI, Pittsburgh Sleep Quality Index; PHQ-9, Patient Health Questionnaire; SDISS, Sheehan Disability Scale; STAI, State-Trait Anxiety Inventory; SOL, sleep onset latency. Bold values are significant ones (p < 0.05).*

None of participants have reported complaints such as discomfort or pain during the examinations.

### Objective and Subjective Sleep Onset Latency

**FIGURE 3:**
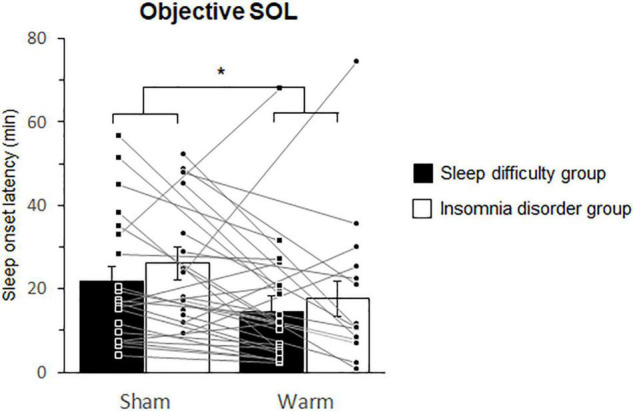
Comparisons of the objective sleep onset latencies between the sham and warm conditions. SOL. Bars and error bars represent the mean ± standard error of the mean (SEM). SOL, sleep onset latency. *p < 0.05.

The Shapiro-Wilk normality tests indicated normal distributions of the objective (*W* = 0.895, *p* = 0.094) and subjective (*W* = 0.917, *p* = 0.100) SOL data. The Levene’s tests also indicated equalities of variances of the objective (*p* = 0.487) and subjective (*p* = 0.791) SOL data. Thus, the objective and subjective SOLs were analyzed using a two-way ANOVA with “group” and “condition” as factors, and a summary of the results is shown in Table 2. There was a significant main effect of condition on objective SOL. *Post hoc* analyses of the effect of condition effect on the objective SOL indicated a significantly shorter SOL in the warm condition compared with the sham condition (Figure 3).

**TABLE 2:** Summary of the statistical results in the objective and subjective sleep onset latency analyses.

Variable	Main effect				Interaction	
	Group		Condition		Group × Condition	
	F	P	F	p	F	p
Objective SOL	0.68	0.417	5.85	0.021*	0.03	0.854
Subjective SOL	0.03	0.863	0.06	0.805	2.41	0.129

*Two-way repeated-measures ANOVAs with group (SLD and INS groups) and condition (sham and warm).*

*ANOVA, analysis of variance; SOL, sleep onset latency; SLD, sleep difficulty; INS, insomnia disorder.*

*p < 0.05.

*Bold values are significant ones (p < 0.05).*

### Changes in Skin Temperature and Distal-Proximal Skin Temperature Gradient

**FIGURE 4:**
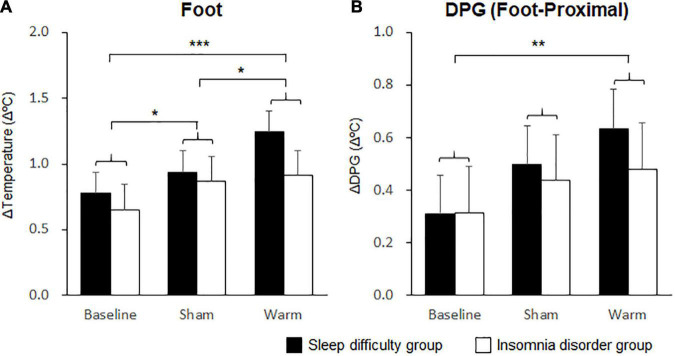
Comparisons of foot skin temperature and distal-proximal skin temperature gradient (DPG) between foot and proximal skin temperatures. **(A)** The mean changes in foot skin temperature over 30 min. **(B)** The mean changes in DPG between the foot and proximal skin temperature over 30 min. Bars and error bars represent the mean ± standard error of the mean (SEM). *p < 0.05, **p < 0.01, ***p < 0.001.

**FIGURE 5:**
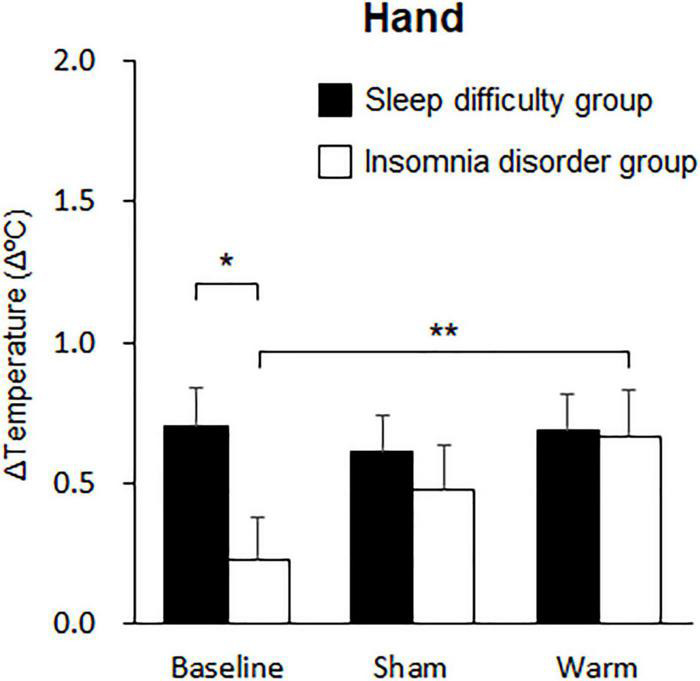
Comparisons of changes in hand skin temperature over 10 min. The black bars represent the sleep difficulties group, and the white bars represent the insomnia disorder group. Bars and error bars represent the mean ± standard error of the mean (SEM). *p < 0.05, **p < 0.01.

Changes in the skin temperature and DPG during sleep were analyzed using mixed-model three-way rANOVAs with “group” and “condition” as factors. The statistical values are summarized in Table 3. The results revealed a significant main effect of condition on both the foot skin temperature and on the DPG between the foot and proximal skin temperatures during the initial 30 min. *Post hoc* analysis for the effect of condition on foot temperature indicated a significantly higher temperature in the warm condition (1.08 ± 0.13°C) than at baseline (0.71 ± 0.13°C; *p* < 0.001; Figure 4A) and in the sham condition (0.90 ± 0.13°C; *p* = 0.04; Figure 4A). *Post hoc* analysis for the effect of condition on the DPG between the foot and proximal skin temperatures indicated a significantly higher temperature in the warm condition (0.56 ± 0.12°C) than at baseline (0.31 ± 0.11°C; *p* = 0.004; Figure 4B). Furthermore, there was a significant interaction between group and condition for the hand skin temperature. *Post hoc* analysis for the interaction indicated a significantly higher temperature in the warm condition (0.67 ± 0.16°C) than at baseline (0.23 ± 0.16°C; *p* = 0.008; Figure 5) within the INS group. There were no significant differences in the hand skin temperature between the three experimental conditions within the SLD group. At baseline, the hand skin temperature was significantly higher in the SLD group (0.71 ± 0.13°C) than in the INS group (0.23 ± 0.16°C; *p* = 0.022; Figure 5).

**TABLE 3:** Summary of the statistical results in the changes of skin temperature analyses.

	Main effect						Interaction							
	Group		Condition		Time		G × C		G × T		C × T		G × C × T	
	F	*p*	*F*	*p*	*F*	*p*	*F*	*p*	*F*	*p*	*F*	*p*	*F*	*p*
Hand	0.15	0.237	2.60	0.076	**77.17**	** < 0.001**	**3.28**	**0.039**	2.05	0.131	0.66	0.619	1.61	0.172
Foot	0.55	0.461	**12.34**	** < 0.001**	**46.10**	** < 0.001**	1.74	0.176	0.42	0.863	0.49	0.921	0.18	0.999
DPG-H	1.46	0.236	2.72	0.068	**42.19**	** < 0.001**	2.09	0.125	1.37	0.256	0.60	0.660	1.56	0.186
DPG-F	0.11	0.741	**5.45**	**0.005**	**11.02**	** < 0.001**	0.55	0.575	0.13	0.992	0.34	0.982	0.12	1.000

*Three-way repeated-measures ANOVAs with group (SLD and INS groups), condition (baseline, sham, and warm), and time as factors for the thermoregulatory variables. ANOVA, analysis of variance; G, group; C, condition; T, time; DPG, distal-proximal skin temperature gradient; DPG-H, DPG between hand and proximal skin temperatures; DPG-F, DPG between foot and proximal skin temperatures; SLD, sleep difficulty; INS, insomnia disorder.*

*Bold values are significant ones (p < 0.05).*

### Heart Rate Variability

**FIGURE 6:**
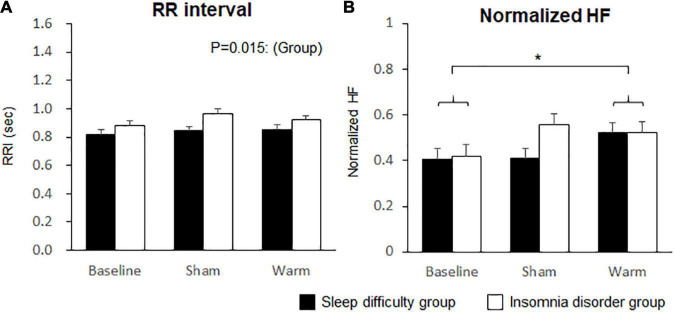
Comparisons of heart rate variability between conditions. **(A)** RR interval. **(B)** Normalized high frequency (HF). Bars and error bars represent the mean ± standard error of the mean (SEM). *p < 0.05.

HRV parameters for the initial 5 min during sleep were analyzed using a two-way ANOVA with “group” and “condition” as factors (Table 4). The results revealed a significant main effect of group on the RR interval (Figure 6A) and a significant main effect of condition on the normHF. *Post hoc* analysis of the effect of condition indicated a significantly higher normHF in the warm condition (0.52 ± 0.03) than at baseline (0.41 ± 0.3; *p* = 0.038; Figure 6B).

**TABLE 4:** Summary of the statistical results in the heart rate variability analyses.

Variable	Main effect				Interaction	
	Group		Condition		Group × Condition	
	*F*	*p*	*F*	*p*	*F*	*p*
RR interval	6.62	0.015	2.01	0.143	0.794	0.457
HF	0.71	0.404	0.45	0.642	0.25	0.778
normHF	1.29	0.260	**3.32**	**0.043**	2.05	0.137

*Two-way repeated-measures ANOVAs with group (SLD and INS groups) and condition (baseline, sham, and warm) as factors for the heart rate variability variables.*

*ANOVA, analysis of variance; normHF, normalized high frequency based on heart rate variability; LF/HF, the ratio of low frequency to high frequency based on heart rate variability; SLD, sleep difficulty; INS, insomnia disorder. Bold values are significant ones (p < 0.05).*

### Contribution of Thermoregulatory Variables and Heart Rate Variability to Sleep Onset Latency

A multiple linear regression analysis was conducted to identify the physiological variables associated with subjective and objective SOLs. The thermoregulatory variables (the DPG between the hand and proximal skin temperatures and the DPG between the foot and proximal skin temperatures) and the cardiac autonomic nervous function (normHF and the LF/HF ratio) were included in the original model. The results of the backward stepwise multiple regression analysis (*R*^2^ = 0.046, *p* = 0.02) indicated that the DPG between the foot and proximal skin temperatures was the main determinant of objective SOL (β = –0.215, *F* = 5.55). The DPG between the hand and proximal skin temperatures, normHF, and LF/HF ratio were not included in the regression model.

## Discussion

In the present study, we investigated the effect of periocular skin warming on sleep onset in individuals with sleep difficulty and insomnia disorder in their home environment. We found that periocular skin warming before bedtime shortened the objective SOL during the nocturnal sleep period in independently of the group. Foot skin temperature and the DPG between the foot and proximal skin temperature were higher in the periocular skin warming condition than that at baseline in independently of the group. In addition, the normalized HF of the HRV was higher in the periocular skin warming condition than at baseline. Furthermore, the reduction of objective SOL associated with periocular skin warming was strongly associated with physiological heat dissipation *via* the foot skin.

### Impact of Periocular Skin Warming on Sleep Onset Latency

We previously reported that periocular skin warming has a favorable effect on subjective sleep initiation in adults with mild difficulty falling asleep (15). Similarly, in the present study, warming the periocular skin region shortened the objective SOL not only in individuals with sleep difficulty but also in patients with insomnia disorder. These results suggest that periocular skin warming using a warming eye mask is effective in promoting sleep onset. Periocular skin warming can be easily and safely performed using an eye mask with an HSG-sheet, and thus, has potential as a novel non-pharmacological therapy for those with insomnia.

### Physiological Heat Dissipation and Sleep Regulation

The increase in the DPG, which is an indicator of physiological heat dissipation *via* distal skin regions, has been found to be more strongly correlated with SOL than the reduction of CBT (14). In the present study, periocular skin warming increased the temperature of the foot skin region and elevated the DPG between the foot and proximal skin region in individuals with sleep difficulty and insomnia disorder, which is comparable with our previous findings in daytime measurements (16, 17). This result suggests that periocular skin warming might facilitate heat dissipation from the distal foot skin region in individuals with sleep difficulty as well as in patients with insomnia disorder. However, it is unclear how periocular skin warming modulates heat dissipation from the foot skin. In one study, Van Someren hypothesized that an increase in skin temperature may act as an input signal to modulate neuronal activity in sleep-regulating brain areas in the hypothalamus and increase sleep propensity (29). In a positron-emission tomography study, Egan et al. demonstrated that afferent signals associated with changes in skin temperature (in the torso, upper arms, and legs) activated the thermoregulatory region of the brain in humans (30). Taken together with these previous results, the present findings suggest that warming of the periocular skin region directly acts as an input signal for modulation of the brain area involved in thermoregulatory function to facilitate heat dissipation from the foot skin region.

### Possible Autonomic Interaction Between Heat Dissipation and Sleep Initiation

Our previous findings suggested that periocular warming elicits a comfortable feeling during the sleep onset period (31). Comfortable stimuli are known to reduce somatic tension (32) and attenuate cutaneous sympathetic nerve activity (33), whereas uncomfortable stimuli increase cutaneous sympathetic nerve activity and reduce distal blood flow (34–36). Relaxation techniques, which are adopted as a therapeutic component of cognitive behavioral therapy for insomnia (32, 37), are effective for the treatment of insomnia and can promote relaxation and sympathetic inhibition (38). Along with a feeling of comfort, periocular skin warming has been reported to enhance cardiac parasympathetic nervous activity and attenuate cardiac sympathetic nervous activity (31). Thus, the facilitation of sleep initiation could be a consequence of enhanced parasympathetic tone or attenuated sympathetic tone. Our backward stepwise multiple linear regression analysis revealed that heat dissipation from the foot region was significantly associated with objective SOL, but that the changes in cardiac autonomic activities were not. Previously, it has also been reported that HRV was not associated with sleep initiation (39). Thus, although periocular skin warming can elicit a comfortable feeling and enhance parasympathetic tone, a reduction in the SOL induced by periocular warming might occur primarily through heat dissipation from distal skin regions.

### Possible Pathophysiology of Insomnia Disorder in Thermoregulation

We also found that periocular skin warming enhanced hand skin temperature only in patients with insomnia disorder. van den Heuvel et al. have hypothesized that patients with insomnia may have impaired heat dissipation from the distal skin when attempting to sleep (40). Heat dissipation capacity from the hands (especially the fingers) is reduced in patients with insomnia compared with healthy adults, while there was no difference in heat dissipation capacity from the foot (40). Collectively, these results suggest that periocular skin warming might reactivate the attenuated heat dissipation from the hand in patients with insomnia disorder. The multiple linear regression model did adopt the DPG between the foot and proximal skin region instead of the DPG between the hand and proximal skin region to predict the objective SOL. This suggests that heat dissipation from the foot skin region more strongly contributes to sleep initiation than that of the hand skin region. Furthermore, diminished heat dissipation from the hand skin region might be feature of the pathophysiology of insomnia disorders. Further studies are needed to elucidate the contribution of diminished heat dissipation from the hand skin region in insomnia pathophysiology and assess the treatment advantage of periocular skin warming in patients with insomnia disorder relative to those with sleep difficulty.

### Limitations

This study has several limitations that should be noted. First, this study was designed as a single-blind trial. Second, the present study was conducted in the participants’ home environment to evaluate the effect of periocular skin warming on sleep in their habitual sleep environment. Thus, the sleep environment, including room temperature between baseline, sham, and warm conditions, could not be strictly controlled. Third, we solely focused on the symptom of difficulty sleep initiation, which means that the effect of periocular warming on the other insomnia symptoms, including daytime dysfunctions, remains unclear. Forth, the objective SOL in the present study was estimated using the original definition of the SLEEP SCOPE system, which defines sleep latency as the time from bedtime to the onset of the first 10 continuous 30-s epochs of sleep. However, sleep latency obtained with the devise has been reported to be longer than that obtained with polysomnography (PSG) (41). Therefore, it may be necessary to evaluate the objective SOL using PSG in future studies. Fifth, according to the clinical guideline for pharmacological treatment (42), the clinical significance threshold for SOL is 10 min, while the decrease of SOL was documented to be 8.5 min in warm condition relative to the sham condition in the present study. As the decrease in SOL by periocular warming did not reach the clinical significance level of treatments of insomnia disorder, it may be necessary to examine the relationship between the duration of warming in the periocular region and improvement of SOL by periocular skin warming in the future study. Sixth, participants were aged between 45 and 70 years, which is the core age group of patients with insomnia disorder. However, most of the previous studies have examined the thermoregulatory responses to skin warming in younger age groups (43). Since the thermoregulatory responses to skin warming has been reported to attenuate with increasing age (44), the effects of periocular skin warming on sleep and body temperature in younger patients with insomnia disorder should be investigated in future studies.

## Data Availability Statement

The raw data supporting the conclusions of this article will be made available by the authors, without undue reservation.

## Ethics Statement

The studies involving human participants were reviewed and approved by the Ethics Committee of Shiga University of Medical Science. The patients/participants provided their written informed consent to participate in this study.

## Author Contributions

TI, AK, TY, and KK contributed to the conception and designed of the study, analyzed the data, and wrote the manuscript. AK, KN, YK, AF, and AY contributed to the clinical assessment. All authors contributed to the article and approved the final version of the manuscript.

## Conflict of Interest

TI was employed by Kao Corporation. AY was employed by Shiga University of Medical Science. The others were employed by Shiga University of Medical Science. AK, KN, TY, and KK were employed by National Center of Neurology and Psychiatry, Japan. Although the funder was not involved in the conduction of experiments and decision to publish, the funder was involved in data analysis and the writing of this article. AY received honoraria for giving lectures from Otsuka Pharmaceutical, Sumitomo Dainippon Pharma, Viatris Pharma, MSD, Janssen Pharmaceutical, Yoshitomi Pharmaceutical Industries, Meiji Seika Pharma. TY received honoraria for giving lectures from MSD and Takeda Pharmaceutical. KK received research support from Eisai, Meiji Seika Pharma, MSD, Otsuka Pharmaceutical, Pfizer, Shionogi, Kao Corporation, PMC, and Takeda Pharmaceutical, and has received honoraria for giving lectures from Eisai, Meiji Seika Pharma, MSD, and Takeda Pharmaceutical. The remaining authors declare that the research was conducted in the absence of any commercial or financial relationships that could be construed as a potential conflict of interest.

## Publisher’s Note

All claims expressed in this article are solely those of the authors and do not necessarily represent those of their affiliated organizations, or those of the publisher, the editors and the reviewers. Any product that may be evaluated in this article, or claim that may be made by its manufacturer, is not guaranteed or endorsed by the publisher.
